# Lipoprotein(a) predicts recurrent cardiovascular events in patients with prior cardiovascular events post-PCI: five-year findings from a large single center cohort study

**DOI:** 10.1186/s12959-022-00424-9

**Published:** 2022-11-21

**Authors:** Na Xu, Yi Yao, Lin Jiang, Jingjing Xu, Huanhuan Wang, Ying Song, Yuejin Yang, Bo Xu, Runlin Gao, Jinqing Yuan

**Affiliations:** Department of Cardiology, National Clinical Research Center for Cardiovascular Diseases, State Key Laboratory of Cardiovascular Disease, Fu Wai Hospital, National Center for Cardiovascular Diseases, Chinese Academy of Medical Sciences and Peking Union Medical College, No 167, Beilishi Road, Xicheng District, Beijing, 100037 China

**Keywords:** Lipoprotein(a), Prior cardiovascular events, Recurrent CVEs

## Abstract

**Background:**

It is well established that lipoprotein(a)[Lp(a)] play a vital role in atherosclerosis. Whether Lp(a) can predict recurrence of cardiovascular events (CVEs) in prior CVEs patients is still unclear. We aim to investigate its association with subsequent long-term adverse events in this high-risk population.

**Methods:**

A total of 4,469 patients with prior CVEs history after PCI were consecutively enrolled and categorized according Lp(a) values of < 10 (low), 10 to 30 (medium), and ≥ 30 mg/dL (high). The primary endpoint was MACCE, a composite of all-cause death, myocardial infarction, stroke and unplanned revascularization.

**Results:**

During an average of 5.0 years of follow-up, 1,078 (24.1%) and 206 (4.6%) patients experienced MACCE and all-cause death with 134 (3.0%) of whom from cardiac death. The incidence of MACCE, all-cause death and cardiac death were significantly higher in the high Lp(a) group (*p* < 0.05). After adjustment of confounding factors, high Lp(a) level remained an independent risk factor for MACCE (adjusted HR 1.240, 95%CI 1.065–1.443, *p* = 0.006), all-cause death (adjusted HR 1.445, 95%CI 1.023–2.042, *p* = 0.037) and cardiac death (adjusted HR 1.724, 95%CI 1.108–2.681, *p* = 0.016). This correlation remained significant when treated as a natural logarithm-transformed continuous variable. This finding is relatively consistent across subgroups and confirmed again in two sensitivity analyses.

**Conclusions:**

Our present study confirmed that Lp(a) was an independent predictor for recurrent CVEs in patients with established CVEs, illustrating that Lp(a) level might be a valuable biomarker for risk stratification and prognostic assessment in this high-risk population.

**Supplementary Information:**

The online version contains supplementary material available at 10.1186/s12959-022-00424-9.

## Introduction

Coronary heart disease (CHD) is a common cardiovascular disease and is a main cause of disability and death. Patients with history of cardiovascular events (CVEs) entail heavy coronary atherosclerotic burden. Despite significant advances in diagnosis and management have improved prognosis of CVEs in the last decades, patients continue to experience CVEs and remain at high-risk for recurrence [1] . Moreover, previous studies reported that healthcare costs of subsequent CVEs are substantial and incremental costs remain elevated for several years after an event [2]. Therefore, identifying predictive biomarkers that would contribute to effective screening or early diagnosis in this high-risk population may reduce recurrent CVEs.

More recently, an increased Lipoprotein(a) [Lp(a)] was identified as a major cardiovascular lipid-related residual risk factor. Lp(a) consists of a low-density lipoprotein (LDL)-like particle that is bound to apolipoproteinB100(apoB), which is then linked with apolipoprotein(a)[apo(a)] [3]. Large prospective epidemiological studies confirmed that Lp(a) is an independent and causal risk factor for a variety of atherothrombotic disorders, most notably CHD through proatherogenic, prothrombotic and proinflammatory effects, it is also related with occurrences of peripheral arterial disease, myocardial infarction(MI), and ischemic stroke [4–6]. Moreover, our previous researches indicated that Lp(a) levels were strongly associated coronary severity and could increase risk of CVEs in patients with three-vessel coronary artery disease (CAD) or chronic kidney disease after percutaneous coronary intervention (PCI) [7–9].

To the best of our knowledge, its clinical implications have not been investigated in the setting of prior CVEs with advanced atherosclerotic burden. Therefore, we aim to evaluate whether Lp(a) is able to predict long-term CVEs recurrence in patients with established CVEs after PCI, based on the analysis from a real-world, prospective, observational cohort of Chinese patients.

## Methods

### Study population

**Fig. 1:**
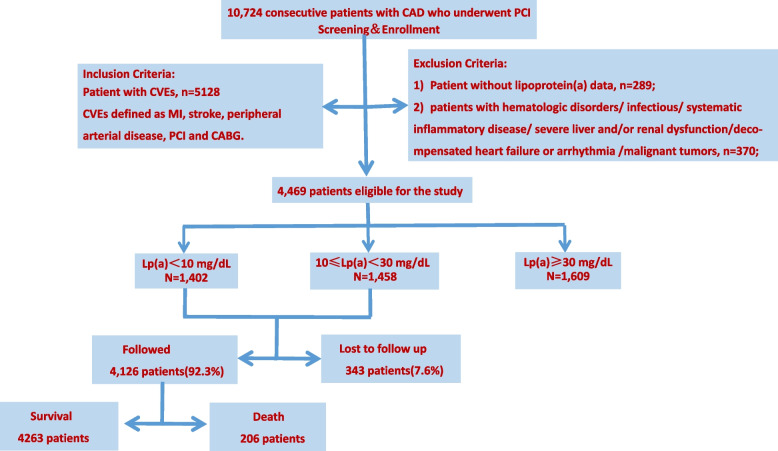
Study flow-chart. Lp(a) = lipoprotein(a), CVEs = cardiovascular events. MI = myocardial infarction; PCI = percutaneous coronary intervention; CABG = coronary artery bypass grafting

This study was based on a prospective, observational, single-center cohort. From January 2013 to December 2013, 10,724 CAD patients were consecutively enrolled undergoing PCI at Fuwai Hospital, Chinese Academy of Medical Sciences (Beijing, China). The study flow-chart was shown as Fig. 1. The inclusion criteria were patients who had experienced a history of prior CVEs [defined as MI, stroke, peripheral arterial disease, PCI and coronary artery bypass grafting (CABG)] before admission. The exclusion criteria were patients with significant hematologic disorders and infectious or systematic inflammatory disease; severe liver and/or renal dysfunction; decompensated heart failure or arrhythmia; malignant tumors, and patients with missing Lp(a) data. Finally, a total of 4,469 patients were enrolled in the current study. Finally, a total of 4,469 patients were enrolled in analysis. The study protocol was approved by the Institutional Review Board of Fuwai Hospital and complied with the Declaration of Helsinki. All patients provided written informed consent before the intervention.

### Laboratory analysis

When admission, all patients are required to take their venous blood after fasting for at least 12 h. Using the automated biochemical analyzer to achieve the concentration measurements of relevant indicators, which including low-density lipoprotein cholesterol (LDL-C), the serum total cholesterol (TC), triglyceride (TG) and high-density lipoprotein cholesterol (HDL-C) (The specific model is Hitachi 7150, Tokyo, Japan). Same as our previous researches [7–9], Lp(a) levels were assayed by an immunoturbidimetry method according to the manufacturer's guide, a latex turbidimetric method [LASAY Lp(a) auto; SHIMA laboratories] and presented in mg/dL with a normal value of < 30 mg/dL. All other laboratory measurements were conducted at the biochemistry center of FuWai Hospital by standard biochemical techniques.

### Procedural details

As described in our previous study [7, 8], the PCI strategy and stent type were left to the discretion of the operating surgeon. Before the procedure, selected patients with PCI who were not on long-term aspirin and/or P2Y12 inhibitors received oral administration of aspirin 300 mg and clopidogrel (loading dose of 300 mg). Patients with acute coronary syndrome (ACS) who were scheduled for PCI received the same dose of aspirin and clopidogrel as soon as possible. During the procedure, unfractionated heparin (100 U/kg) was administered to all of the patients, and glycoprotein IIb/IIIa inhibitors were used according to the operator’s judgment. More than 50% stenosis of the left main artery, left anterior descending artery, left circumflex artery, right coronary artery, and main branch of these vessels was defined as coronary artery stenosis. More than 70% stenosis of these vessels was indicated for coronary stent implantation. After the procedure, aspirin was prescribed at a dose of 100 mg daily indefinitely, and clopidogrel 75 mg daily or ticagrelor 90 mg twice daily for at least 1 year was recommended after PCI.

### Patient follow-up

All of the patients were evaluated at five time points after the discharge (1, 6 and 12-month, and 2-year, and thereafter up to 5-year). Follow-up data were collected through medical records, telephone calls, or clinical visits. Investigator training, blinded questionnaire filling, and telephone recording were performed to achieve high-quality data. The primary endpoint was major adverse cardiovascular and cerebrovascular events (MACCE), of which all-cause death, MI, unplanned revascularization and stroke during the follow-up were included. The secondary outcomes included all-cause death and cardiac death. Death that could not be attributed to a noncardiac etiology was considered cardiac death. As for myocardial infarction, the third general definition is chosen as its definition in this paper [10]^.^ Unplanned revascularization specifically refers to the treatment of target vessels of ischemic symptoms and events by means of repetitive percutaneous access or surgical bypass. Stroke was defined as a loss of neurological function with residual symptoms at least 72 h after onset [11].

### Statistical analysis

Continuous variables are reported as mean ± standard deviation or median with interquartile range and categorical variables were presented as number (percentage). Comparisons between continuous variables were performed by independent sample Student’s t test or Mann–Whitney U test and the chi-square test for categorical variables. The Lp(a) was subsequently analyzed as categorical variable, patients were stratified into three groups according to Lp(a) distribution [Low Lp(a)(< 10 mg/dL), Medium Lp(a)(10 mg/dL ≤ Lp(a) < 30 mg/dL), High Lp(a) (30 ≥ mg/dL)]. Survival curves were constructed by the Kaplan–Meier method and compared by the log-rank test according to Lp(a) groups. Univariable and multivariable Cox proportional hazard regressions were performed to calculate the hazard ratio (HR) and 95% confidence interval (CI) and evaluate the associations between Lp(a) levels (as a categorical or log-transformed continuous variable) and clinical outcomes. The multivariable model was adjusted for the following covariates in an all-enter way: age, sex, ACS, family history of CAD, hypertension, diabetes, smoking, SYNTAX score, Left ventricular eject fraction (LVEF), estimated glomerular filtration rate (eGFR), high sensitivity C-reactive protein (hsCRP), TG, LDL-C, statins because of their statistical significance in univariate analysis or clinical importance. Restricted cubic spline curves (RCS) were created to assess linearity assumptions of the relationship between Lp(a) and MACCE and all-cause death.

Exploratory subgroup analyses of the primary outcome were performed according to age (< 65 or ≥ 65 years), sex (male or female), diabetes (yes or no), hypertension(yes or no), presentation (SAP or ACS), statins (yes or no), LDL-C leves (< 1.8 or ≥ 1.8 mmol/L) and hsCRP levels(≤ 3 or > 3 mg/L) between plasma Lp(a) level (< 30 or ≥ 30 mg/dL) and these covariates were tested to interpret potential subgroup differences. The above-described multivariable Cox proportional hazards models were used for the interaction and subgroup analyses.

Meanwhile, we performed sensitivity analysis of to confirm the association of plasma Lp(a) concentration for risk prediction of MACCE by 2 methods, which excluding subjects with Lp(a) levels in the top or the bottom 5% and excluding participants with prior CABG, stroke and peripheral arterial disease. The prevalence of all-cause mortality is 2% of the population in Beijing, China. The present desired significance level was *P* < 0.05, and the study power was 0.80, thus, the overall required sample size was calculated to be 3000 cases to be able to detect statistical significance. Two-sided *p*-value of < 0.05 were considered statistically significant. Analyses were performed using SPSS software version 25.0 (IBM Corporation, Chicago, IL) and R language (version 3.5.2, Feather Spray; The R Foundation for Statistical Computing, Vienna, Austria).

## Results

### Baseline Characteristics

**Fig. 2:**
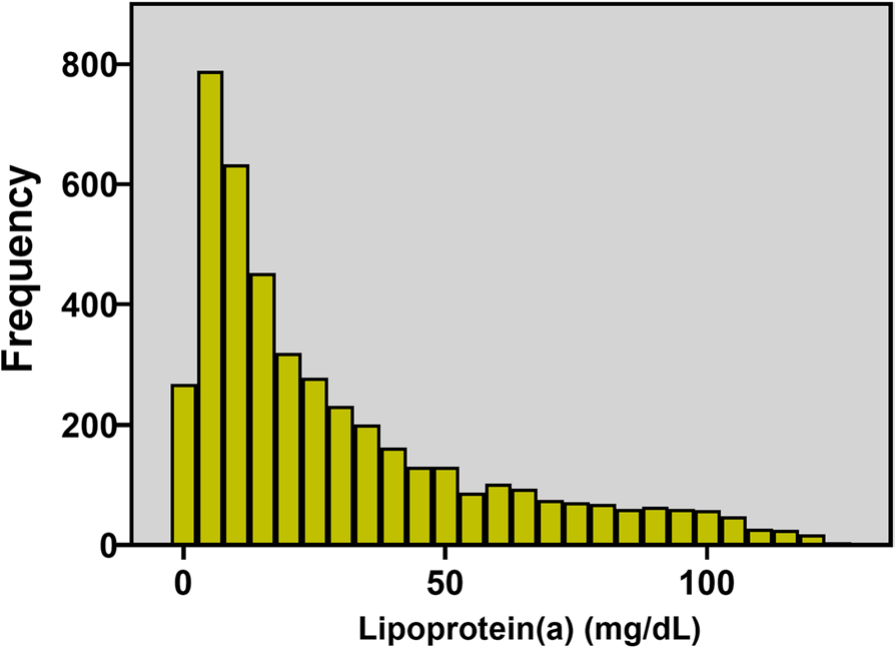
Distribution of lipoprotein(a) levels in the study population

As presented in Fig. 1, A total of 4,469 patients eligible were ultimately included in the study. According to Lp(a) concentrations, patients were divided into three subgroups as low Lp(a) group (< 10 mg/dL, *n* = 1,402), medium Lp(a) group (≥ 10 and < 30 mg/dL, *n* = 1,458), high Lp(a) group (≥ 30 mg/dL,*n* = 1,609), respectively. In the overall population, Lp(a) levels had a skewed distribution with a tail toward the highest levels, which was consistent with previous researches [12] (Fig. 2).

The baseline clinical and laboratory characteristics of the study participants were shown in Table 1. In general, the mean age of the study was 59.51 ± 10.27 years, and 3,577(80.0%) were male. Participants in high Lp(a) group were more older and female, with higher TC, LDL-C, but lower plasma TG, LVEF, BMI, and tend to have lower proportion of current smoker. The baseline glucose, HbA1c and eGFR did not differ significantly among the three groups. Notably, baseline Lp(a) and hsCRP levels were significantly elevated from low to high Lp(a) subjects (*p* < 0.001). Additionally, the prevalence of cardiovascular risk profiles such as hypertension, dyslipidemia, diabetes, family history of CAD and chronic obstructive pulmonary disease(COPD) did not differ significantly across the three groups. Moreover, the prevalence of left main disease and STNTAX score were similar among groups. With regard to secondary prevention medication, there was no significant differences in the proportion of aspirin, P2Y12 inhibitor, statins, ACEI/ARB, β-blockers, and CCB among groups.

**Table 1:** Baseline clinical, angiographic and medication according to Lp(a) categories

	**All patients (*n*=4,469)**	**Lp(a) categories(mg/dl)**			***p*** **value**
		**<10 (*n*=1,402)**	**10-30 (*n*=1,458)**	**≥30 (*n*=1,609)**	
	***n*=8,550**	***n*=950**	***n*=3,714**	***n*=3,886**	
Age, yrs	59.51±10.27	59.15±10.22	59.30±10.42	60.03±10.16	0.041
Sex, male(%)	3577(80.0)	1163(83.0)	1166(80.0)	1248(77.6)	0.001
BMI, kg/m2	25.98±3.11	26.15±3.10	26.00±3.12	25.83±3.08	0.018
Risk factors					
Hypertension	3034(67.9)	954(68.0)	975(66.9)	1105(68.7)	0.559
Hypercholesterolemia	3202(71.6)	1024(73.0)	1042(71.5)	1136(70.6)	0.329
Diabetes mellitus	1507(33.7)	499(35.6)	468(32.1)	540(33.6)	0.140
Current smoker	2722(60.9)	886(63.2)	906(62.1)	930(57.8)	0.005
Family history of CAD	1114(24.9)	335(23.9)	349(23.9)	430(26.7)	0.189
COPD	117(2.6)	29(2.1)	38(2.6)	50(3.1)	0.205
Laboratory findings					
HbA1c,%	6.30(5.90-7.20)	6.30(5.90-7.20)	6.30(5.90-7.10)	6.30(5.90-7.20)	0.277
Glucose, mmol/L	6.32±2.26	6.39±2.23	6.30±2.37	6.28±2.18	0.324
LVEF, %	61.15±8.15	61.77±7.88	60.80±8.29	60.93±8.23	0.003
Hs-CRP, mg/L	1.52(0.76-3.55)	1.36(0.73-2.83)	1.58(0.74-3.92)	1.69(0.82-4.04)	< 0.001
eGFR, ml/min	89.64±16.02	90.22±15.51	89.49±16.61	89.28±15.89	0.248
HDL-C, mmoL/L	1.02±0.28	1.01±0.28	1.01±0.27	1.05±0.28	< 0.001
LDL-C, mmoL/L	2.41±0.89	2.25±0.84	2.37±0.88	2.57±0.91	< 0.001
TG, mmoL/L	1.75±1.03	1.94±1.29	1.70±0.96	1.61±0.78	< 0.001
TC, mmoL/L	4.08±1.05	3.96±1.03	4.02±1.04	4.23±1.06	< 0.001
Lp(a), mg/dl	18.93(7.90-43.03)	5.06(3.13-7.43)	17.36(13.08-23.14)	55.69(40.18-79.75)	< 0.001
Presentation					0.045
ACS	2284(51.1)	702(50.1)	784(53.8)	798(49.6)	
Stable angina	2185(48.9)	700(49.9)	674(46.2)	811(50.4)	
Left main involvement	126(2.8)	36(2.6)	37(2.5)	53(3.3)	0.355
Mean SYNTAX score	11.46±8.54	11.17±8.37	11.48±8.55	11.69±8.68	0.259
Medication at discharge					
aspirin	4407(98.6)	1384(98.7)	1436(98.5)	1587(98.6)	0.873
clopidogrel	4397(98.4)	1379(98.4)	1439(98.7)	1579(98.1)	0.465
ACEI/ARB	2561(57.3)	803(57.3)	828(56.8)	930(57.8)	0.852
Statin	4256(95.2)	1334(95.1)	1386(95.1)	1586(95.5)	0.859
β-blocker	4071(91.1)	1260(89.9)	1327(91.0)	1484(92.2)	0.076
CCB	2121(47.5)	668(47.6)	660(45.3)	793(49.3)	0.083

Continuous values are summarized as mean±SD, median (Q1-Q3) and categorical variables as n (percentage)

*Lp(a)* = lipoprotein(a), *BMI* = body mass index, *CAD* = coronary artery disease *COPD* = chronic obstructive pulmonary disease; *HbA1c* = glycated hemoglobin; *LVEF* = left ventricular ejection fraction; *Hs-CRP* = high-sensitivity C-reactive protein; *eGFR* = estimated glomerular filtration rate; *HDL-C* = high density lipoprotein cholesterol; *LDL-C* = low density lipoprotein cholesterol; *TG* = triglyceride; *TC* = total cholesterol; *ACS* = acute coronary syndrome; *ACEI/ARB* = angiotensin converting enzyme inhibitor/angiotensin receptor blockers; *CCB* = calcium channel blockers

### Relation of Risk factors and Recurrent Adverse Events

The distribution of clinical parameters with and without MACCE was summarized in Supplementary Table 1. Compared to those free of events, those with MACCE were older, with higher levels of baseline glucose, HbA1c and hsCRP level, but with lower LVEF and estimated GFR. The proportion of hypertension and diabetes were significantly higher in patients who developed MACCE. The mean concentration of TG, TC, LDL-C and HDL-C did not differ significantly between the groups. Of note, MACCE patients had higher STNTAX score compared with the controls (*p* = 0.005). While, there was no significant difference between the two groups in the presence rate of dyslipidemia, family history of CAD, smoking and COPD, as while as proportion of Left main involvement and ACS presentation. Moreover, there was no difference in terms of medication prescriptions between the event and non-event ones. Specially, the MACCE-present group had statistically higher admission Lp(a) levels than the MACCE-absent group [21.56 (8.6.2–46.98) vs. 18.12 (7.68–41.72) mg/dL, *p* = 0.002].

### Lipoprotein(a) and Recurrent Adverse Events

The overall median follow-up time was 5.0 years (interquartile range 3.0–5.1 years), and the response rate was 92.3% (Fig. 1). During the period, 1,078 (24.1%) and 206 (4.6%) patients experienced MACCE and all-cause death with 134 (3.0%) of whom from cardiac death. In total cohort, the incidence of MACCE in low, medium and high Lp(a) group was 22.1%, 22.4% and 27.4%, based on the cut-off value of 10 and 30 mg/dL, respectively.

**Fig. 3:**
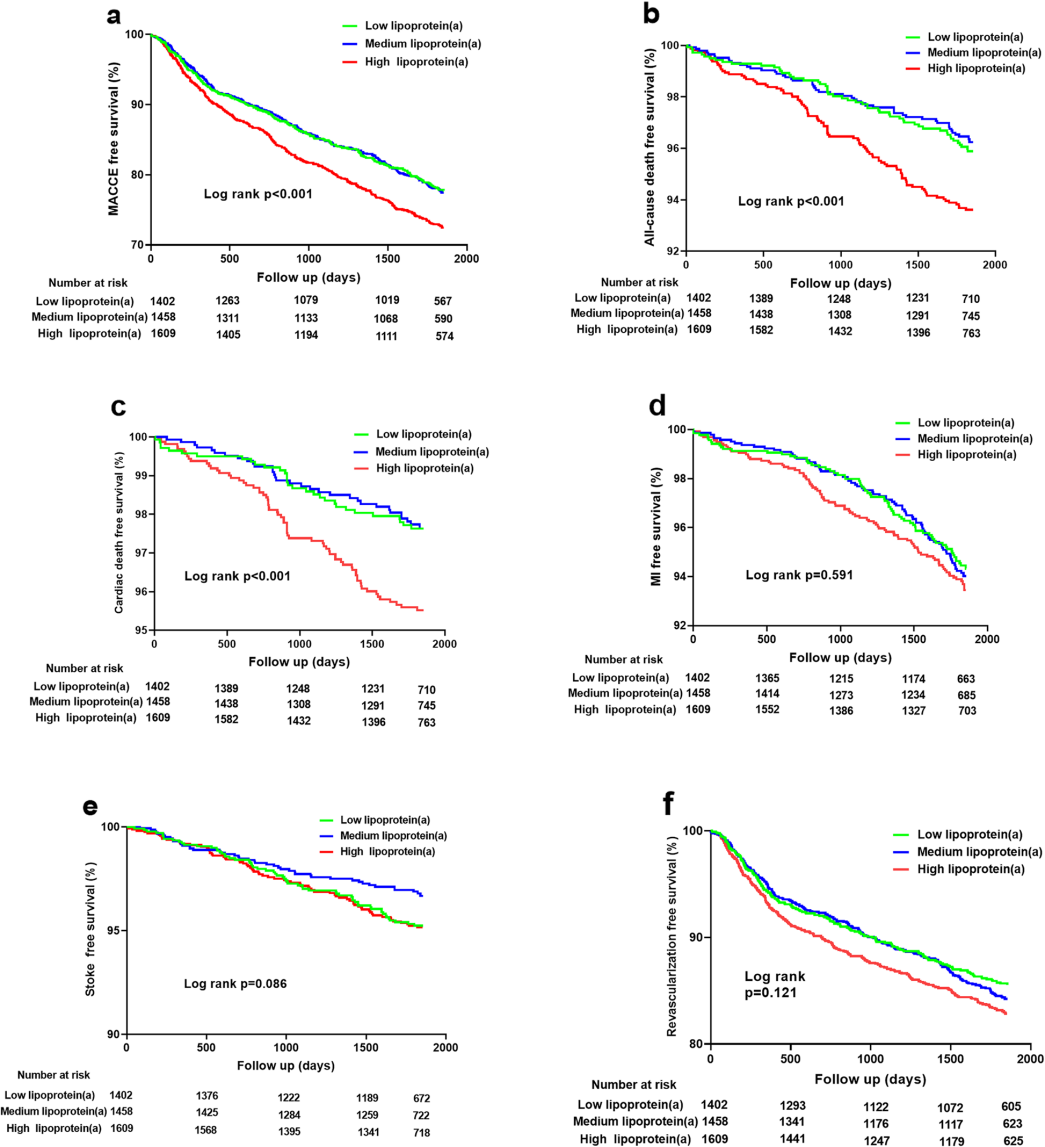
Cumulative incidence curves for primary and secondary endpoints according to different Lp(a) levels. (**a**-**f**) Cumulative incidences of MACCE (**a**), all-cause death (**b**), cardiac death(**c**), myocardial infarction (**d**), stroke (**e**), and unplanned revascularization (**f**)

**Fig. 4:**
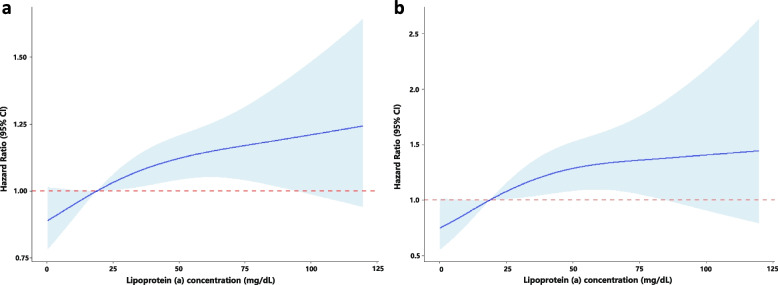
Restricted cubic spline curves (RCS) for the relationship between Lp(a) and MACCE (**a**) and all-cause death (**b**)

Significantly increased risks of MACCE, all-cause death, and cardiac death occurred in the high Lp(a) group compared with the low Lp(a) group (all *p* < 0.05, Table 2). Of note, the significantly higher risk of MACCE in the higher Lp(a) group was mainly driven by all-cause death, because there were no significant differences among groups in the risk for MI, stroke and revascularization (all p > 0.05). Cumulative free survival of the whole cohort according to Lp(a) subgroups was estimated by Kaplan–Meier curves and revealed similar results (Fig. 3. log-rank *p* < 0.001 for MACCE, all-cause death and cardiac death). Univariable Cox analysis showed that high Lp(a) level was associated with higher risks of MACCE(crude HR 1.239, 95% CI 1.072–1.433, *p* = 0.004), all-cause death (crude HR 1.597, 95% CI 1.146–2.225, *p* = 0.006) and cardiac death (crude HR 1.939, 95%CI 1.269–2.962, *p* = 0.002), but not MI, stroke, or unplanned revascularization (Table 2). After adjustment for covariates, high Lp(a) level remained an independent risk factor for MACCE (adjusted HR 1.240, 95% CI 1.065–1.443, *p* = 0.006), all-cause death (adjusted HR 1.445, 95% CI 1.023–2.042, *p* = 0.037) and cardiac death (adjusted HR 1.724, 95% CI 1.108–2.681, *p* = 0.016). When modeled as a continuous variable per unit increase in log-transformed Lp(a) concentration, multivariate Cox regression analysis showed an independent association that high Lp(a) group had 1.140-fold risk of MACCE (95%CI 1.006–1.292, *p* = 0.040), 1.386-fold risk of all-cause death (95%CI 1.026–1.872, *p* = 0.033) and 1.591-fold risk of cardiac death (95%CI 1.087–2.330, *p* = 0.017) after adjustment. RCS showed a nonlinear relation between Lp(a) on continuous scales and the risk of MACCE and all-cause death (Fig. 4).

**Table 2:** Association of Lp(a) levels with recurrent cardiovacular outcomes in patients with prior CVEs during 5-year follow-up

Endpoints	Events, n/total no.(%)	Crude HR (95% CI)	Crude *p-*value	Adjusted HR (95% CI)	Adjusted *p-*value
MACCE					
Log-Lp(a)	—	1.140 (1.012–1.285)	0.031	1.140 (1.006–1.292)	0.040
Lp(a) < 10	310/1402 (22.1%)	1.00 (reference)	—	1.00 (reference)	—
10 ≤ Lp(a) < 30	327/1458 (22.4%)	1.012 (0.866–1.182)	0.884	1.029 (0.878–1.207)	0.720
Lp(a) ≥ 30	441/1609 (27.4%)	1.239 (1.072–1.433)	0.004	1.240 (1.065–1.443)	0.006
All-cause daeth					
Log-Lp(a)	—	1.506 (1.132–2.002)	0.005	1.386 (1.026–1.872)	0.033
Lp(a) < 10	54/1402 (3.9%)	1.00 (reference)	—	1.00 (reference)	—
10 ≤ Lp(a) < 30	53/1458 (3.6%)	0.942 (0.645–1.376)	0.757	0.891 (0.605–1.312)	0.559
Lp(a) ≥ 30	99/1609 (6.2%)	1.597 (1.146–2.225)	0.006	1.445 (1.023–2.042)	0.037
Cardiac death					
Log-Lp(a)	—	1.776 (1.236–2.552)	0.002	1.591 (1.087–2.330)	0.017
Lp(a) < 10	31/1402 (2.2%)	1.00 (reference)	—	1.00 (reference)	—
10 ≤ Lp(a) < 30	34/1458 (2.3%)	1.053 (0.647–1.713)	0.836	1.001 (0.609–1.646)	0.996
Lp(a) ≥ 30	69/1609 (4.3%)	1.939 (1.269–2.962)	0.002	1.724 (1.108–2.681)	0.016
Myocardial infarction					
Log-Lp(a)	—	1.122 (0.894–1.409)	0.321	1.138 (0.897–1.445)	0.287
Lp(a) < 10	86/1402 (6.1%)	1.00 (reference)	—	1.00 (reference)	—
10 ≤ Lp(a) < 30	94/1458 (6.4%)	1.048 (0.782–1.404)	0.752	1.096 (0.812–1.479)	0.548
Lp(a) ≥ 30	115/1609 (7.1%)	1.165 (0.881–1.540)	0.285	1.190 (0.887–1.595)	0.245
Stroke					
Log-Lp(a)	—	0.875 (0.661–1.159)	0.352	0.843 (0.627–1.133)	0.257
Lp(a) < 10	62/1042 (4.4%)	1.00 (reference)	—	1.00 (reference)	—
10 ≤ Lp(a) < 30	45/1458 (3.1%)	0.696 (0.474–1.022)	0.064	0.715 (0.484–1.056)	0.092
Lp(a) ≥ 30	72/1609 (4.5%)	1.012 (0.720–1.421)	0.947	0.965 (0.676–1.379)	0.847
Revascularization					
Log-Lp(a)	—	1.116 (0.960–1.298)	0.152	1.155 (0.986–1.353)	0.074
Lp(a) < 10	192/1402 (13.7%)	1.00 (reference)	—	1.00 (reference)	—
10 ≤ Lp(a) < 30	218/1458 (15.0%)	1.089 (0.897–1.322)	0.390	1.126 (0.923–1.374)	0.241
Lp(a) ≥ 30	262/1609 (16.3%)	1.189 (0.987–1.432)	0.069	1.241 (1.021–1.508)	0.030

*Lp(a)* Lipoprotein(a), *CVEs* Cardiovascular events, *MACCE* Major adverse cardiovascular and cerebrovascular events, *HR* Hazard ratio, *CI* Confidence interval

*p* values < 0.05 indicate statistical significance

**Fig. 5:**
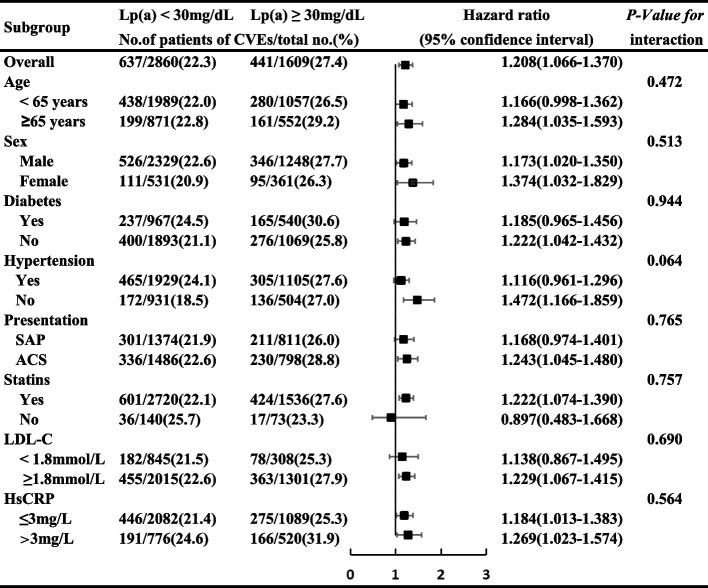
Subgroup analyses for the primary endpoint. HRs and 95% CIs were calculated by reference to the Lp(a) < 30 mg/dL group. The interaction between Lp(a) and each covariate was tested by a multivariable Cox proportional hazards regression model

The relationship of higher Lp(a) level (< 30 or ≥ 30 mg/dL) with MACCE risk was relatively consistent across the subgroups of age, sex, diabetes, hypertension, presentation, baseline statins use, LDL-C levels and hsCRP levels(Fig. 5). There were no significant interactions between high Lp(a) level and these covariates (interaction *p* > 0.05 for all subgroups).

In a sensitivity analysis by excluding population with Lp(a) levels in the top or the bottom 5%(*n* = 447), revealed that high Lp(a) was considered to be a statistically risk factor of MACCE (adjusted HR 1.253, 95%CI 1.062–1.479, *p* = 0.007). In addition, another sensitivity analysis which excluding subjects with CABG, stroke and peripheral arterial disease (*n* = 1686), showed that high Lp(a) remains an independent predictor of MACCE in this population (adjusted HR 1.238, 95%CI 1.016–1.509, *p* = 0.035) after adjusting for potential confounding factors (shown in Supplementary Table 2).

## Discussion

This study is conducted on a sizable high-risk population with prior CVEs to investigate an association between higher baseline Lp(a) levels and recurrent CVEs. Our study demonstrated that high plasma Lp(a) level is an independent risk factor for 5-year MACCE, all-cause death, and cardiac death and is relatively consistent across subgroups. This finding was confirmed again in two sensitivity analyses. The present study illustrates that Lp(a) level might be a valuable biomarker for risk stratification and prognostic assessment in prior CVEs population.

Cardiovascular disease(CVD) have been widely established as the leading cause of premature morbidity and mortality in China [13]. Results of subanalysis of the NAGOYA HEART Study(NHS) reported that subsequent composite CVE incidence in patients with previous CVD was approximately 3.5 greater than patients without CVD [14]. Consequently, the substantial clinical burden of prior CVE patients urgently need a novel risk factors that mediate residual risk for early diagnosis, integrated treatment and targeted intervention.

Circulating Lp(a) level is a genetically determined risk factor and remain relatively constant throughout a person’s lifetime [15]. Evidence from observational and genetic studies support a causal role of Lp(a) in the development of CAD, including CHD and peripheral arterial disease, as well as ischemic stroke [3, 16]. Effect sizes are most pronounced for MI and peripheral arterial disease where Lp(a) concentrations predict 2- to threefold increases in risk. Moreover, several evidences from secondary prevention studies also presented that elevated Lp(a) level is significantly and independently associated with subsequent CVEs during follow-up after occurrence of CVEs including individual history of PCI, CABG, MI, stroke and peripheral arterial disease [17–21]. However inconsistent findings from other studies have been reported that elevated Lp(a) have no impact on atherothrombotic events in population with established CVD [22, 23]. Thus, further exploration of the association between Lp(a) and future cardiovascular outcomes is warranted to provide more evidence-based information, especially for high-risk populations such as history of composite CVEs.

Epidemiological studies and guidelines proposed that CVD risk is associated with increasing Lp(a) levels > 30 mg/dl (> 75 nmol/l) in primary care populations in a dose dependent fashion [24], thereby we further divided the population into three groups based on Lp(a) levels of 10 and 30 mg/dL. Ultimately, in the present study with large sample size and long duration of follow-up, we discovered that elevated Lp(a) either as a categorical or a continuous variable remained an independent risk factor for MACCE. Our findings are consistent with those of a previous study [12], which recruited 2,284 diabetes patients with prior CVEs, showed that Lp(a) was an independent predictor for recurrent CVEs in diabetes patients with prior CVEs. Additionally, the association between Lp(a) and incidences of MACCE were further confirmed in two sensitivity analyses. Moreover, because Lp(a) levels differed between individuals of genders and age, and the prognostic value of Lp(a) may affected by LDL-C level, hsCRP levels and comorbidities states. The subgroup analyses were performed and turned out to be consistent across the different subgroups. Concurrently, we also found that Lp(a) ≥ 30 mg/dL was significantly associated with greater risk of all-cause mortality and cardiac death. A similar trend was found in a large cohort study, which studied individuals from two prospective studies of the Danish general population [25], of which 6,976 had information on Lp(a) concentrations. Observationally, Lp(a) > 93 mg/dL group were associated with a hazard ratio of 1.50 (95% CI1.28–1.76) for cardiovascular mortality and of 1.20 (1.10–1.30) for all-cause mortality, not for non-cardiovascular mortality compared with Lp(a) < 10 mg/dL. However, Given the data mainly conducted in healthy participant of the general population rather than patients with prior CVE. Future secondary prevention studies are warranted to clarify these results.

Plasma Lp(a) levels contributes to the poor prognosis in prior CVEs patients may through proatherogenic, prothrombotic and proinflammatory mechanisms [26–28]. The true role of elevated Lp(a) levels in the setting of secondary prevention is a profoundly important issue, given that this patient population is at high risk of recurrent CVEs and has higher Lp(a) levels than general population. The phase 3 clinical trial is on the horizon, which assess the impact of Lp (a) lowering with antisense oligonucleotide therapies directed against apolipoprotein(a) expression on CVEs in patients with CVD, may address this issue [29]. In conclusion, our present study further extended the association of Lp(a) with long-term cardiovascular outcomes in the setting of established CVEs with heavy atherosclerotic burden, and Lp(a) measurement may help to further risk stratification for this high-risk population.

### Limitations

There were several strengths of our study, including the large sample size, extended follow-up to 5-year duration, the high follow‐up rate, adequate adjustment for potential confounders and performed analyses on subgroups. However, several limitations that should be noticed in the present study. First, single measurement of Lp(a) was only tested at admission, the level of Lp(a) may change over follow-up was not available. Second, Lp(a) was measured by immunoturbidimetry method in the current study, its accuracy might be influenced by the apo(a) size isoform-dependent bias, Lp(a) assay standardization is needed [30]. Meanwhile, effect of sample handling and storage on Lp(a) measurement should be considered. Last, this was an observational study that may be subject to potential selection biases. More high-quality researches are required to address the impact of Lp(a)-lowing on poor prognosis of high-risk population in secondary prevention.

## Supplementary Information

**Additional file 1:** **Supplementary Table 1**. Baseline clinical, angiographic and medication of the study patients with and without MACCE at 5 yeas.

**Additional file 2:** **Supplementary Table 2**. Relation of Lp(a) levels with prior CVEs patients at 5-year MACCE in Sensitivity Analyses.

## Data Availability

Due to ethical restrictions related to the consent given by subjects at the time of study commencement, our datasets are available from the corresponding author upon reasonable request after permission of the Institutional Review Board of Fuwai Hospital.
