# Supplementary material for: Lipoprotein(a) predicts recurrent cardiovascular events in patients with prior cardiovascular events post-PCI: five-year findings from a large single center cohort study
Source: Thromb J. 2022 Nov 21;20:69. doi: 10.1186/s12959-022-00424-9 (PMC9682694; doi:10.1186/s12959-022-00424-9)
Supplement: Supplementary file 1 — Additional file 1: Supplementary Table 1. Baseline clinical, angiographic and medication of the study patients with and without MACCE at 5 yeas. [file 12959_2022_424_MOESM1_ESM.docx]

**Supplementary Table 1 Baseline clinical, angiographic and medication of the study patients with and without MACCE at 5 yeas.**

| **Variables** | **Without MACCE (n=3,391 )** | **With MACCE (n=1,078 )** | ***P*-value** |
| --- | --- | --- | --- |
| Age, yrs | 59.24 ± 10.23 | 60.38 ± 10.34 | 0.001 |
| Sex, male(%) | 2705 (79.8%) | 872 (80.9%) | 0.423 |
| BMI, kg/m2 | 25.98 ± 3.09 | 26.01 ± 3.16 | 0.781 |
| Risk factors |  |  |  |
| Hypertension | 2264 (66.8) | 770 (71.4) | 0.004 |
| Hypercholesterolemia | 2418 (71.3) | 784 (72.7) | 0.367 |
| Diabetes mellitus | 1105 (32.6) | 402 (37.3) | 0.004 |
| Current smoker | 2058 (60.7) | 664 (61.6) | 0.596 |
| Family history of CAD | 851 (25.1) | 263 (24.4) | 0.840 |
| COPD | 81 (2.4) | 36 (3.3) | 0.089 |
| Laboratory findings |  |  |  |
| HbA1c,% | 6.30 (5.90-7.10) | 6.40 (5.90-7.40) | 0.001 |
| Glucose, mmol/L | 6.25 ± 2.17 | 6.55 ± 2.49 | < 0.001 |
| LVEF, % | 61.38 ± 7.96 | 60.44 ± 8.70 | 0.001 |
| Hs-CRP, mg/L | 1.47 (0.74-3.34) | 1.69 (0.84-4.21) | < 0.001 |
| eGFR, ml/min | 90.13 ± 15.71 | 88.11 ± 16.86 | < 0.001 |
| HDL-C, mmoL/L | 1.02 ± 0.28 | 1.02 ± 0.28 | 0.485 |
| LDL-C, mmoL/L | 2.40 ± 0.89 | 2.44 ± 0.88 | 0.222 |
| TG, mmoL/L | 1.74 ± 1.04 | 1.77±1.01 | 0.459 |
| TC, mmoL/L | 4.07±1.05 | 4.11 ± 1.05 | 0.245 |
| Lp(a), mg/dl | 18.12 (7.68-41.72) | 21.56 (8.62-46.98) | 0.002 |
| Presentation |  |  | 0.292 |
| ACS | 1718 (50.7) | 566 (52.5) |  |
| Stable angina | 1673 (49.3) | 512 (47.5) |  |
| Left main involvement | 94 (2.8) | 32 (3.0) | 0.734 |
| Mean SYNTAX score | 11.26 ± 8.30 | 12.10 ± 9.22 | 0.005 |
| Medication at discharge |  |  |  |
| aspirin | 3343 (98.6) | 1064 (98.7) | 0.775 |
| clopidogrel | 3337 (98.4) | 1060 (98.3) | 0.861 |
| ACEI/ARB | 1942 (57.3) | 619 (57.4) | 0.930 |
| Statin | 3231 (95.3) | 1025 (95.1) | 0.790 |
| β-blocker | 3093 (91.2) | 978 (90.7) | 0.624 |
| CCB | 1601 (47.2) | 520 (48.2) | 0.557 |

Continuous values are summarized as mean±SD, median (Q1-Q3) and categorical variables as n (percentage).

Lp(a) = lipoprotein(a); BMI = body mass index; CAD = coronary artery disease; COPD = chronic obstructive pulmonary disease; HbA1c = glycated hemoglobin; LVEF = left ventricular ejection fraction; Hs-CRP = high-sensitivity C-reactive protein; eGFR = estimated glomerular filtration rate; HDL-C = high density lipoprotein cholesterol; LDL-C =low density lipoprotein cholesterol; TG = triglyceride; TC = total cholesterol; ACS =acute coronary syndrome; ACEI/ARB = angiotensin converting enzyme inhibitor/angiotensin receptor blockers; CCB =calcium channel blockers.
