# Supplementary material for: Lipoprotein(a) predicts recurrent cardiovascular events in patients with prior cardiovascular events post-PCI: five-year findings from a large single center cohort study
Source: Thromb J. 2022 Nov 21;20:69. doi: 10.1186/s12959-022-00424-9 (PMC9682694; doi:10.1186/s12959-022-00424-9)
Supplement: Supplementary file 2 — Additional file 2: Supplementary Table 2. Relation of Lp(a) levels with prior CVEs patients at 5-year MACCE in Sensitivity Analyses. [file 12959_2022_424_MOESM2_ESM.docx]

**Supplementary Table 2 Relation of Lp(a) levels with prior CVEs patients at 5-year MACCE in Sensitivity Analyses**

| Category | Crude HR  (95% CI) | Crude  *p-*value | Adjusted HR  (95% CI) | Adjusted  *p-*value |
| --- | --- | --- | --- | --- |
| Sensitivity analysis 1 |  |  |  |  |
| Lp(a)＜10 | 1.00 (reference) | — | 1.00 (reference) | — |
| 10≤Lp(a)＜30 | 1.037 (0.881-1.222) | 0.661 | 1.062 (0.899-1.256) | 0.479 |
| Lp(a)≥30 | 1.255 (1.070-1.470) | 0.005 | 1.253 (1.062-1.479) | 0.007 |
| Sensitivity analysis 2 |  |  |  |  |
| Lp(a)＜10 | 1.00 (reference) | — | 1.00 (reference) | — |
| 10≤Lp(a)＜30 | 1.032 (0.847-1.258) | 0.753 | 1.075 (0.877-1.317) | 0.485 |
| Lp(a)≥30 | 1.226 (1.016-1.479) | 0.034 | 1.238 (1.016-1.509) | 0.035 |

In sensitivity analysis 1, subjects with lipoprotein(a) levels in the top or the bottom 5% were excluded (n=447).

In sensitivity analysis 2, subjects with prior CABG, stroke and peripheral arterial disease were excluded (n=1,686).

Lp(a) = lipoprotein(a), CVEs = cardiovascular events, MACCE = major adverse cardiovascular and cerebrovascular events.

HR = hazard ratio, CI = confidence interval.
